# On the mechanism of solid-state phase transitions in molecular crystals – the role of cooperative motion in (quasi)racemic linear amino acids

**DOI:** 10.1107/S2052252520001335

**Published:** 2020-02-27

**Authors:** M. M. H. Smets, E. Kalkman, A. Krieger, P. Tinnemans, H. Meekes, E. Vlieg, H. M. Cuppen

**Affiliations:** a Radboud University, Institute for Molecules and Materials, Heyendaalseweg 135, 6525 AJ Nijmegen, The Netherlands

**Keywords:** polymorphs, single-crystal-to-single-crystal phase transitions, quasi-racemates, classical nucleation theory

## Abstract

During single-crystal-to-single-crystal (SCSC) phase transitions, a polymorph of a compound can transform to a more stable form while remaining in the solid state. By understanding the mechanism of these transitions, strategies can be developed to control this phenomenon. This is particularly important in the pharmaceutical industry, but also relevant for other industries such as the food and agrochemical industries. Although extensive literature exists on SCSC phase transitions in inorganic crystals, it is unclear whether their classications and mechanisms translate to molecular crystals, with weaker interactions and more steric hindrance. A comparitive study of SCSC phase transitions in aliphatic linear-chain amino acid crystals, both racemates and quasi-racemates, is presented. A total of 34 transitions are considered and most are classified according to their structural change during the transition. Transitions without torsional changes show very different characteristics, such as transition temperature, enthalpy and free energy, compared with transitions that involve torsional changes. These differences can be rationalized using classical nucleation theory and in terms of a difference in mechanism; torsional changes occur in a molecule-by-molecule fashion, whereas transitions without torsional changes involve cooperative motion with multiple molecules at the same time.

## Abstract 2

A comparative study of 34 single-crystal-to-single-crystal phase transitions in aliphatic linear-chain amino acid crystals is applied to obtain insight into their transition mechanism.

## 1. Introduction   

Polymorphism is the ability of a compound to exist in more than one crystal structure form, a very common phenomenon (Bernstein, 2002 ▸). This results in different physico–chemical properties such as colour, melting point, solubility and dissolution rate. Polymorphic compounds can exhibit solid-state phase transitions, for example, where one polymorph can transform into a more stable form. By understanding the mechanism of solid-state phase transitions, strategies can be developed to control the polymorph obtained. This is particularly important in the pharmaceutical industry because the dissolution rate, as well as the formal approval of pharmaceuticals, is often linked to a specific polymorph. Polymorphic control is also relevant in other industries including food and agrochemicals. However, the understanding of solid-to-solid polymorphic transitions in molecular crystals is still in its infancy. Extensive literature exists on phase transitions in inorganic crystals, or crystals with less molecular complexity. It is not evident how the classification and phase-transition mechanism in these materials translate to molecular crystals, which have weaker interactions and more steric hindrance (Brandel *et al.*, 2015 ▸; Herbstein, 2006 ▸; Dunitz, 2016 ▸). Moreover, a number of concepts such as cooperative motion remain controversial (Mnyukh, 2010 ▸).

The study of phase transitions in molecular crystals has recently gained a renewed interest after the report of several examples of dynamic crystals. These crystalline materials respond to external stimuli (*e.g.* exposure to light, pressure or temperature) with mechanical motion such as translation, rotation, jumping, bending and twisting (Naumov *et al.*, 2015 ▸; Commins *et al.*, 2016 ▸; Reddy *et al.*, 2010 ▸).

Particularly interesting are thermosalient – *jumping* – crystals, which are crystals that jump or leap upon heating or cooling. The movement can be many times their own size and is caused by a thermally induced single-crystal-to-single-crystal (SCSC) phase transition. Because of the regular structure of the material, small perturbations in weak interactions can be amplified through collective motion. This collective aspect of the process results in a rapid response in the full material leading to amplified effects. Although the behavior has already been described in 1983 by Etter & Siedle (1983 ▸), interest in these kinds of materials has only recently intensified after the realization of their potential as switchable smart materials. Several cases of jumping crystals have been reported in the literature (Ding *et al.*, 1991 ▸; Davey *et al.*, 1994 ▸; Sahoo *et al.*, 2013 ▸), but a mechanistic and theoretical understanding of the underlying mechanism is lacking, which is essential to further optimize these materials for future applications.

**Figure 1:**
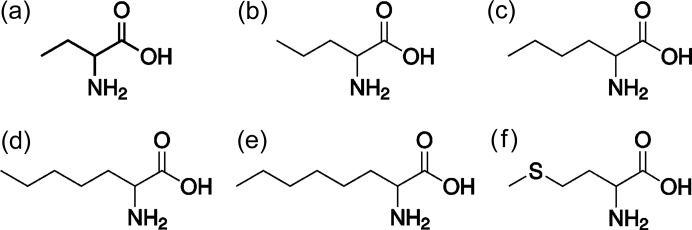
Molecular structures of the amino acids studied: (*a*) 2-aminobutyric acid, (*b*) norvaline, (*c*) norleucine, (*d*) 2-aminoheptanoic acid, (*e*) 2-aminooctanoic acid and (*f*) methionine.

In the present work, we compare solid-state phase transitions in several aliphatic linear-chain amino acids (see Fig. 1 ▸), the racemates dl-2-aminobutyric acid (dl-Abu), dl-norvaline (dl-Nva), dl-norleucine (dl-Nle), dl-2-aminoheptanoic acid (dl-Hep), dl-2-aminooctanoic acid (dl-Oct), dl-methionine (dl-Met) and their mutual quasi-racemates. These amino acids all crystallize in similar packings and all exhibit SCSC phase transitions. Some show thermosalient behavior, whereas others do not. With this comparison, we aim to understand the phase-transition mechanism for this class of materials and to shed more light on the determining factor for thermosalient behavior. For comparison, we verified or improved literature values of phase transition characteristics, such as the transition temperature, enthalpy and entropy. In addition, new crystal structures and phase transition characteristics are included. The phase transitions are first classified into several groups based on the changes in the crystal structures involved, and then their transition enthalpy, transition entropy and hysteresis are compared. We will further present thermal stage microscopy data. We will show that both nucleation-and-growth theory and cooperative motion are compatible with our results, and thus are not mutually exclusive.

## 2. Controversy surrounding cooperativity   

Ehrenfest classified phase transitions as first or second order depending on whether the chemical potential is discontinuous with respect to first or second derivatives of thermodynamic variables (Ehrenfest, 1933 ▸). The Landau theory of phase transitions is based on the description of the chemical potential with respect to the temperature as a power series in an order parameter. This order parameter can, in principle, be used to pin down the order of phase transitions (Landau & Lifshitz, 1980 ▸). In the general Fischer classification, phase transitions are divided into first-order phase transitions and continuous phase transitions of higher order (Fisher, 1967 ▸). Other classifications are mostly based on the structural differences between two polymorphs, *e.g.* the structural classification of Buerger (1961 ▸).

In the 1970s and subsequent decades, Mnyukh *et al.* strongly criticized the existing classifications of solid-state phase transitions, and stated that all solid-state phase transitions proceed through a nucleation-and-growth mechanism and therefore only first-order phase transitions exist (Mnyukh & Petropavlov, 1972 ▸; Mnyukh & Panfilova, 1973 ▸; Mnyukh *et al.*, 1975 ▸; Mnyukh, 2010 ▸). According to Mnyukh (2010 ▸), many phase transitions previously classified as second order have been proven over the years to be first order, and no proven second-order phase transition has been found. The nucleation-and-growth mechanism involves layer-by-layer edge-wise growth, which is described by Mnyukh as probably occurring through molecule-by-molecule structural rearrangement. Mnyukh excludes the possibility of 2D or 3D cooperative motion, because he links this intimately to second-order or continuous phase transitions. In the literature on phase transitions in molecular crystals, the term ‘cooperative motion’ or ‘concerted motion’ is frequently used without specifying its length scale. Here we use these terms for the simultaneous movement of multiple neighboring molecules, on a length scale of tens to a few hundreds of molecules. This is different from cooperative motion in second-order phase transitions with an infinite correlation length, which was suggested by Mnyukh (2010 ▸).

In the literature, thermosalient transitions are often referred to as ‘martensitic’ as a synonym for involving cooperative motion, but these notions are not simply interchangeable. A martensitic transformation is described as a diffusionless displacive phase transition in metals, alloys or ceramics, which occurs very fast – in theory up to the velocity of sound – via cooperative movements of large numbers of atoms over small distances at a coherent, glissile interface. Characteristic for martensitic transformations is that the shape change induced by the displacement is relatively large and dominated by shear, so that the strain energy dominates the kinetics (Zhang & Kelly, 2009 ▸; Callister, 2007 ▸). According to a review by Roitburd and Kurdjumov, a martensitic transformation is a first-order phase transition that ‘proceeds under conditions where the initial phase maintains metastability’, in other words, shows hysteresis and/or spread in transition temperature (Roitburd & Kurdjumov, 1979 ▸). The latter review explains the theory behind structural changes and kinetics of martensitic transformations in great detail, but also acknowledges that the principle behind it is nucleation-and-growth. Since the use of ‘martensitic transformation’ for molecular crystals appears to raise ambiguity in the literature, we have chosen to avoid the term in this context.

In a review by Herbstein, Mnyukh’s theory is recognized as the most likely mechanism for solid-state phase transitions, but it is viewed as a macroscopic description that cannot explain all complexities, especially in molecular crystals (Herbstein, 2006 ▸). Brandel *et al.* summarized the concepts developed over the last two decades for complex effects found in molecular crystals that need further investigation: ‘cooperativity, packing frustration, concerted movements, microstructure and mosaicity, modulated structures, zip-like mechanisms, anomalous thermal expansion, lattice strains, internal molecular motions, mesoscopic effects, *etc.*’ (Brandel *et al.*, 2015 ▸).

## 3. Structural characteristics of aliphatic linear-chain amino acids   

Racemates and quasi-racemates of linear-chain amino acids typically crystallize in multiple possible polymorphs that are enantiotropically related. One speaks of enantiotropically related polymorphs if the relative stability of different polymorphs changes reversibly as a function of temperature.

### 3.1. Racemates   

**Figure 2:**
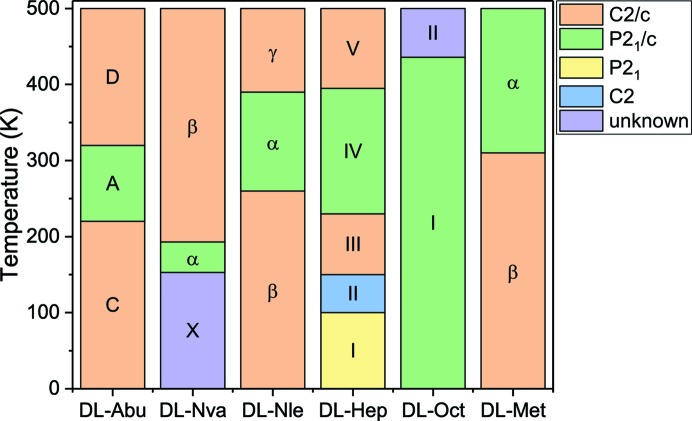
Schematic of the enantiotropically related polymorphs of linear amino acid racemates and their stability temperature interval; space group symmetries are indicated.

**Figure 3:**
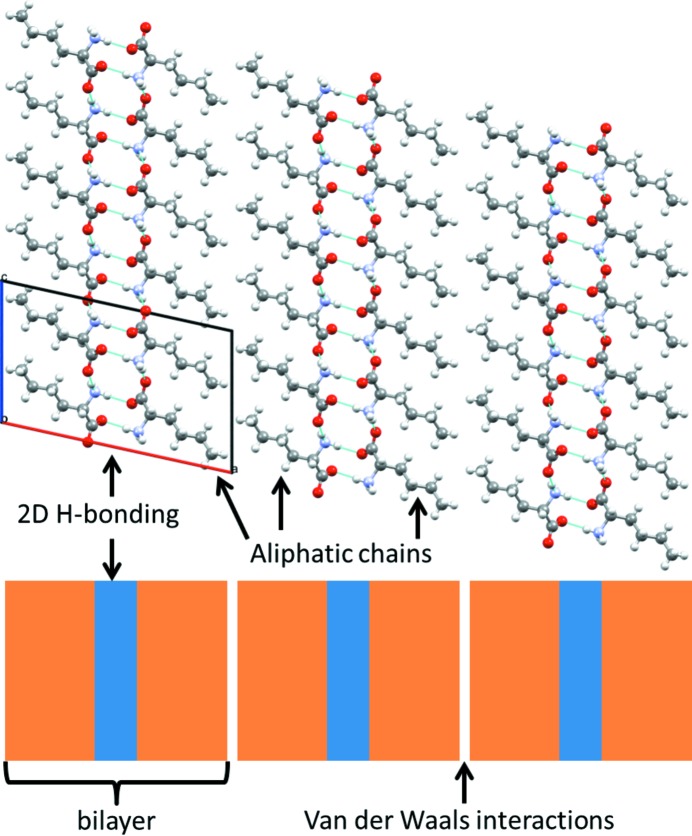
Schematic of the typical crystal structure of the racemic aliphatic linear-chain amino acids; dl-Nle is used as an example.

The enantiotropically related polymorphs of the linear-chain amino acid racemates are shown in Fig. 2 ▸ (Görbitz *et al.*, 2012 ▸, 2014 ▸, 2015 ▸; Görbitz, 2011 ▸; Coles *et al.*, 2009 ▸; Smets *et al.*, 2015 ▸, 2016 ▸, 2017 ▸; Xynogalas, 2003 ▸). In all structures, the molecules are arranged in bilayers that are held together by relatively strong hydrogen bonds between the zwitterionic amino and carboxylic acid groups (see Fig. 3 ▸). Much weaker van der Waals interactions exist between these bilayers, and for most cases the polymorphic transitions involve relative shifts between these bilayers, in some cases accompanied by a torsional change or even disorder in the torsions. Disorder occurs especially for the smaller molecules (dl-Abu forms A and D, and dl-Nva forms α and β) or for high-temperature polymorphs (dl-Hep form V). The hydrogen bonding network remains intact during all phase transitions. For this reason, dl-cysteine was not included in this comparison, since its transitions also involve forming or breaking hydrogen bonds between the sulfur in the aliphatic tail and the hydrogen bonding network (Kolesov *et al.*, 2008 ▸; Minkov *et al.*, 2009 ▸, 2011 ▸). Typically, linear-chain racemic amino acids crystallize in one of the space groups *P*2_1_/*c* or *C*2/*c*, with some exceptions (see Fig. 2 ▸).

### 3.2. Quasi-racemates   

A quasi-racemate is a co-crystal with a 1:1 mixture of two similar compounds of opposite chirality. Often, the crystal structure is similar to the true racemates of the individual components. Görbitz and co-workers have solved the structures of many quasi-racemates of hydrophobic amino acids [see Görbitz & Karen (2015 ▸); Görbitz *et al.* (2016*b* ▸), and references therein]. Many of the crystal structures resemble the linear-chain racemates, except for the quasi-racemate d-Nle:l-Abu, which has a different hydrogen bonding pattern [typically referred to as l1–d1 (Görbitz *et al.*, 2009 ▸)] and shows no solid-state phase transitions. Table 1 ▸ shows the number of enantiotropically related polymorphs found for linear-chain amino acid quasi-racemates with the racemates on the diagonal. The anomalous d-Nle:l-Abu structure is shown in italics. This overview relies on structures from the aforementioned literature as well as new experiments. Here we applied liquid-assisted grinding for crystallization and the crystals obtained were then screened using differential scanning calorimetry (DSC) for transitions. Powder X-ray diffraction was used for confirmation by measuring structural information at temperatures between the phase transition temperatures found in the DSC. Where possible, single-crystal X-ray diffraction was used for full structure determination. For the quasi-racemates d-Abu:l-Met, d-Nva:l-Nle, d-Nva:l-Met and d-Met:l-Nle, literature data were used (Görbitz & Karen, 2015 ▸; Görbitz *et al.*, 2016*b* ▸). The remaining quasi-racemates, d-Abu:l-Nva, d-Abu:l-Hep, d-Nva:l-Hep, d-Nle:l-Hep, d-Met:l-Hep, d-Oct:l-Hep, d-Abu:l-Oct, d-Nva:l-Oct, d-Nle:l-Oct and d-Met:l-Oct, were obtained as part of this study. Newly identified phase transitions are indicated in Table 1 ▸ in bold.

**Figure 4:**
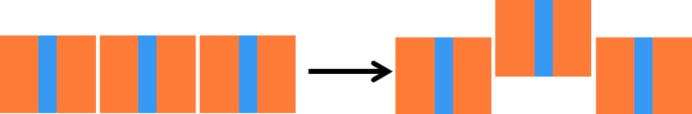
Schematic of the typical bilayer displacement during solid-state phase transitions in linear-chain amino acids.

The SCSC phase transitions of some of the quasi-racemates were investigated in detail, and it was found that two types of phase transition occur (Görbitz & Karen, 2015 ▸). The first type is a phase transition involving only the slide of bilayers (see Fig. 4 ▸), and the second type is a disordering transition that involves torsional changes in the molecules. For the bilayer slide, the phase transitions consist of two consecutive steps: every second bilayer interface slides in the first step, forming an intermediate structure, whereas in the second step (at a slightly higher or lower temperature) the other interfaces slide. During bilayer-slide transitions, the conformation of the molecules remains similar. The associated enthalpy and entropy changes are relatively small. The disordering transitions can be gradual (‘continuous’) or discontinuous, the latter involving relatively large enthalpy and entropy changes.

In the remainder of the paper, we will use the following four categories to classify all transitions: (1) phase transitions that do not involve any torsional change of the molecules, but only relative displacements of bilayers (‘pure shifts’); (2) transitions that also involve a torsional change at the end of the aliphatic chain, which also includes order–disorder transitions (‘outer torsions’); (3) transitions with a change in any other torsional angle of the molecule (‘inner torsions’); and finally (4) phase transitions that are expected to fall in one of the first three categories, but are not well-characterized because the crystal structure of at least one of the polymorphs has not yet been determined.

Görbitz *et al.* (2016*a* ▸) presented an overview of the structures of l-amino acids and their transitions. They found that these involve a number of different processes: alteration of the hydrogen-bond pattern (not considered here), sliding of molecular bilayers (our class 1), side-chain rearrangements, and abrupt as well as gradual modifications of the side-chain disorder (our classes 2 and 3). Sliding of molecular bilayers typically does not lead to a phase transition in l-amino acids, since this results in the same structure, unless the molecules in the bilayer have alternating conformations, like the cases of forms I and III of l-Phe (Cuppen *et al.*, 2019 ▸).

## 4. Thermodynamic characteristics of solid-state phase transitions   

**Figure 5:**
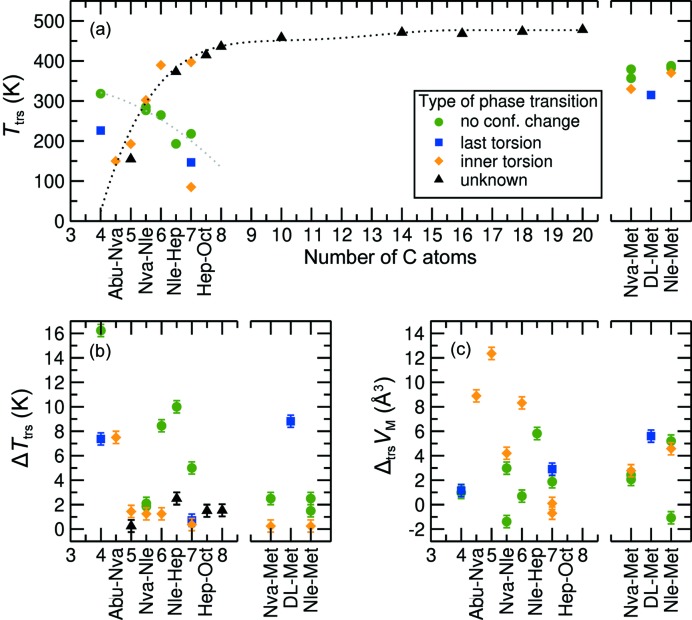
Characteristics of the solid-state phase transitions of linear-chain racemic and quasi-racemic amino acids: (*a*) transition temperatures of the racemates up to a chain length of 20 carbon atoms, and quasi-racemates up to a chain length of 8 carbon atoms; (*b*) hysteresis; and (*c*) transition volume changes versus the number of carbon atoms per molecule. The dotted lines are guides to the eye. For the quasi-racemates (all with Δ*n* = 1) the average chain length was used, *i.e.* 5.5 for d-Nva-l-Nle. All structures involving Met are on the right-hand side of the graph.

Two enantiotropically related polymorphs have equal Gibbs free energy at their phase transition temperatures *T*_trs_,
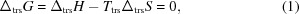
where the equilibrium phase transition parameters are denoted by the subscript trs; Δ_trs_ *H* is the transition enthalpy, *T*_trs_ is the transition temperature and Δ_trs_ *S* is the transition entropy. At a temperature *T*′ close to *T*_trs_, the driving force for a phase transition, Δμ (kJ mol^−1^), is in good approximation proportional to the temperature difference Δ*T* = *T*′ − *T*_trs_,
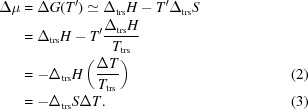
This allows us to estimate the transition entropy and transition driving force from the measured transition enthalpy, transition temperature and measured hysteresis which we assume to be 2Δ*T*. Here we use a large set of related structures of which these thermodynamic and structural parameters are determined in order to understand the SCSC phase transition in detail. Figs. 5 ▸ and 6 ▸ show the estimated transition temperature, hysteresis (2Δ*T*), transition volume changes, transition enthalpy, calculated transition entropy (Δ_trs_ *S* = Δ_trs_ *H*/*T*_trs_) and calculated driving force of the transition (Δμ = |Δ_trs_ *S*Δ*T*|) as a function of carbon chain length. Fig. 5 ▸(*a*) not only contains the transition temperatures of the aforementioned amino acids, but also some values for longer chain racemic amino acids obtained by Xynogalas (2003 ▸), for which the crystal structures have not been determined. The hysteresis in the observed transition temperatures is indicated by the length of the error bars in Fig. 5 ▸(*b*). Since all phase transitions described here involve discrete enthalpy and entropy changes, and hysteresis, they are first order.

**Figure 6:**
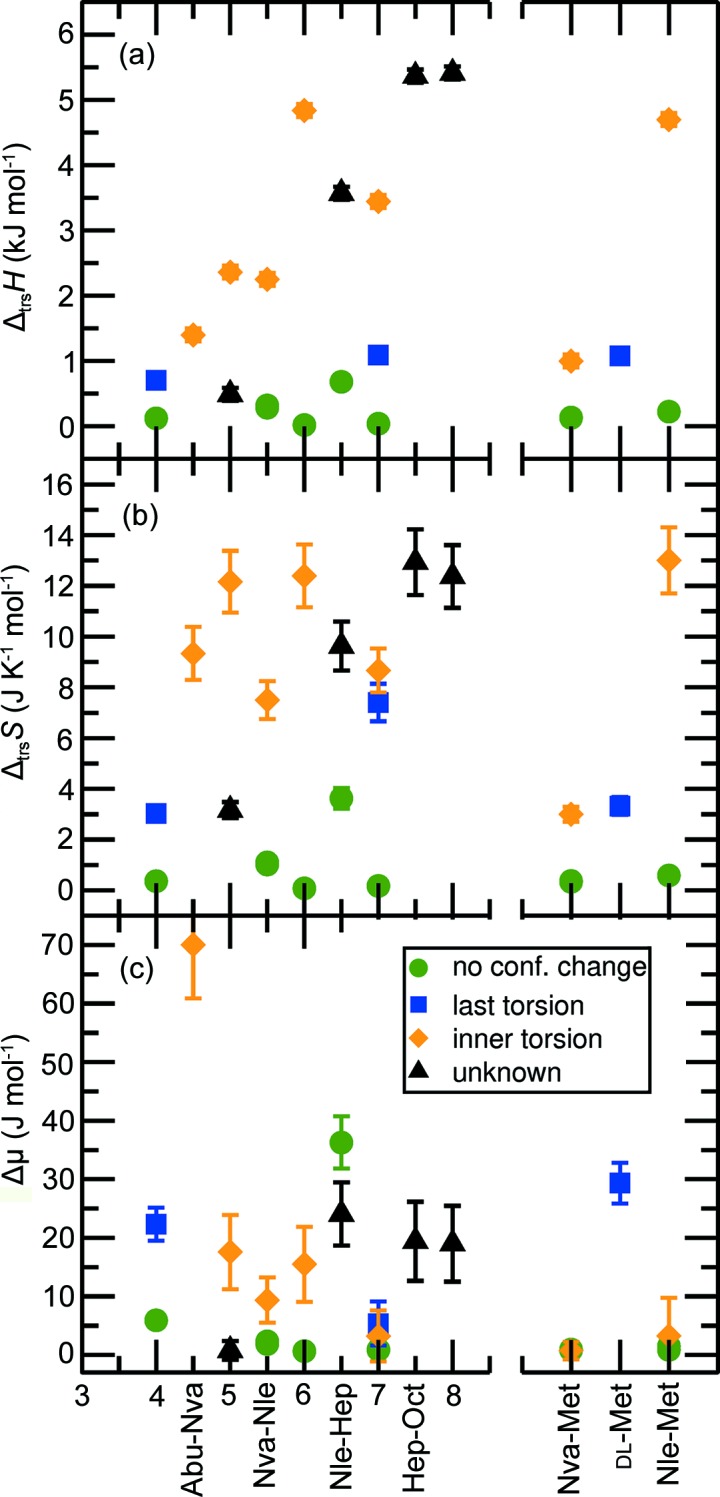
Characteristics of the solid-state phase transitions of linear-chain racemic and quasi-racemic amino acids; (*a*) transition enthalpies, (*b*) transition entropies and (*c*) transition Gibbs free energy [driving force, equation (3)] versus the number of carbon atoms per molecule.

The four categories of solid-state phase transition, as defined in Section 3.2, are distinguished using different symbols and colors as indicated in the legends to Figs. 5 ▸(*a*) and 5 ▸(*c*). Figs. 6 ▸(*a*) and 6 ▸(*b*) show that the enthalpy and entropy changes are generally smallest for the transitions without torsional changes. Transitions that involve large structural changes, typically the case for torsional changes close to the hydrogen bonding layer (inner), have the largest change in enthalpy and entropy, *i.e.* orange > blue > green. Volume changes [Fig. 5 ▸(*c*)] show no clear correlation with the type of phase transition, but shorter (quasi)racemates appear to have larger (absolute) volume changes, which often coincide with a change in disorder in the crystal structures. The transition temperatures on the other hand [Fig. 5 ▸(*a*)] show some clear trends, especially for the ‘inner torsion’ class where the transition temperature increases with chain length (black dotted line). dl-Hep has two transitions within this class: one follows this group, the other is an outlier (around 100 K). The I ↔ II transition is a disorder transition and is rather special since it shows a volume increase upon cooling.

The enthalpy data for the ‘inner torsion’ class show a similar increasing trend. It is very suggestive, based on both graphs, to classify the unknown transitions from the work by Xynogalas (2003 ▸) as transitions involving a torsional change. The increase in transition temperature with chain length is very much in line with what is observed for other aliphatic compounds like fatty acids and alkanes, suggesting an increasing energy barrier for nucleation of the new phase with chain length. However, for the phase transitions without torsional changes the trend is reversed (gray dotted line). We will return to this point later. The two lines cross near the co-crystal d-Nva:l-Nle. For this co-crystal, step-wise transitions have been observed at close temperatures (Görbitz & Karen, 2015 ▸), as mentioned earlier.

Hysteresis [Fig. 5 ▸(*b*)] is a measure for Δ*T* in equation (4), and is used to calculate the driving force for the phase transition, Δμ, where the phase transition is in fact observed. Fig. 6 ▸(*c*) shows that almost all phase transitions without a torsional change occur at a similar very small driving force. The scatter in Δ*T* and Δ_trs_ *S* within this class is large. This means that a phase transition with a larger transition entropy requires less over-heating or -cooling to arrive at the same driving force. Consequently, hysteresis will generally be smaller for phase transitions with a higher transition entropy compared with phase transitions of lower transition entropy. The driving force for the phase transitions with a torsional change is on average larger and has a large spread.

There are a few outliers within the four categories. Firstly, d-Nva:l-Met has a relatively low transition enthalpy and entropy for the second category. This phase transition is spread out over a large temperature regime and entails a gradual increase in disorder of the torsions. Also the phase transition of d-Abu:l-Nva deviates, since it involves a large hysteresis for the third category. Another deviating phase transition is the low-temperature transition of d-Nle:l-Hep, which has a relatively large enthalpy and especially entropy change for a phase transition of the first category. This is probably related to the large volume change during the phase transition, as can be seen in Fig. 5 ▸(*c*).

The unknown low-temperature *X* ↔ α phase transition of dl-Nva at 155 K belongs to the second category phase transitions judging by its Δ_trs_ *H* and Δ_trs_ *S* values; therefore, the unknown low-temperature form *X* is likely to differ only in the last torsion angle from the α form, or only a part of the molecule changes, involving inner torsions. Since the literature indicates that the α conformations might be partly present in the form *X*, it seems likely that the low-temperature form *X* of dl-Nva is a high-*Z*′ structure, similar to the low-temperature forms I and II of dl-Hep, with partial preservation of the conformations of the α form.

When comparing the transition enthalpy and entropy of the unknown phase transition of dl-Oct at 426 K in Fig. 6 ▸, the high values indicate a probable change in at least one torsion angle, but not only the last torsion angle, which is consistent with the NMR data described by Smets (2018 ▸).

## 5. Nucleation theory for phase transitions in a layered system   

The family of amino acids described in this work shows only first-order phase transitions, but spread over a range of transition enthalpies, dependent mainly on the extent of the structural changes. Phase transitions with a very low enthalpy of transition, such as the β ↔ α phase transition of dl-Nle, can be overlooked easily or even wrongly viewed as second-order phase transitions. A second-order phase transition is quite unlikely for these phase transitions with displacements of bilayers, since this involves energy barriers, evident from the hysteresis. Nevertheless, this raises the questions of whether and how to distinguish first-order phase transitions with a low transition enthalpy and similar structures from second-order phase transitions. When the observed transition enthalpy is lower than the margin of error of the DSC, this will be the bottleneck in unequivocally determining whether a phase transition is first or second order. In the layered crystals of the present study, the phase transition is sometimes spread over a large temperature range, because the layers transform independently, and the critical nucleus for each layer is reached in a stochastic fashion. This increases the likelihood of not observing a transition enthalpy, despite the phase transition being first order, because, for example, in a DSC measurement the transition peak is split up into many very small peaks (Smets *et al.*, 2015 ▸). Solid-state NMR measurements of these amino acids appear to be quite sensitive to small differences in crystal structures, especially in the case of the β ↔ α phase transition of dl-Nle (Smets *et al.*, 2015 ▸). Therefore, solid-state NMR could be used to show that a phase transition is first order if the resolution is sufficient to show co-existence of two phases. However, it will be difficult to prove a phase transition is second order using these techniques, since this can only be assumed as a consequence of the absence of evidence for a first-order phase transition. To some extent, discussion of whether a phase transition is first or second order is mainly an academic one, because the presence of impurities and lattice defects obscures possible second-order phase transitions in real systems.

Another factor that complicates the characterisation of phase transitions is that the aliphatic chains of the amino acids described here have a large degree of conformational freedom. At elevated temperatures in particular, the chains display increased thermal motion or disorder that changes gradually with temperature. This clouds the line between increased flexibility, thermal lattice expansion and phase transitions between polymorphs. In the end, a phase transition always involves a change in symmetry (Drebushchak *et al.*, 2011 ▸; Dunitz, 2016 ▸). The gradual changes in disorder in the quasi-racemates especially appear to almost have the characteristics of a second-order phase transition (Görbitz & Karen, 2015 ▸), but the observable transition enthalpy indicates that the phase transitions are first order.

**Figure 7:**
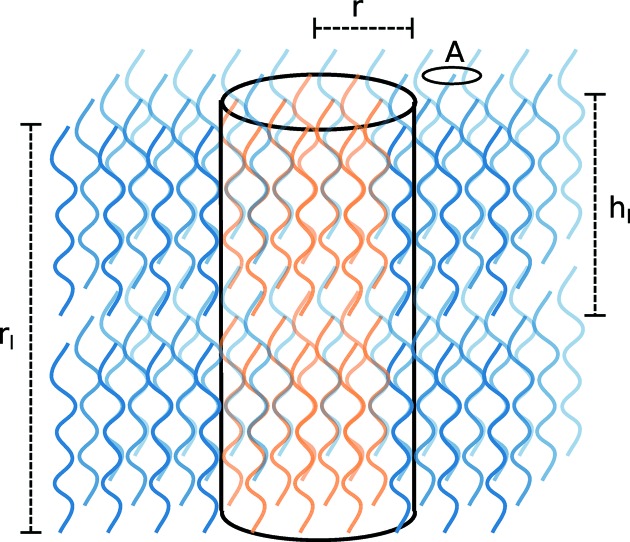
Schematic representation of a nucleus in a layered system. The nucleus is assumed to be cylindrical in shape of height *r*_l_ and radius *r*. The height of an individual layer is *h*_l_ and the area an individual molecule occupies in the layer is *A*.

First-order transitions are generally assumed to proceed through a nucleation-and-growth mechanism. We hence derive an expression for the critical nucleus and free-energy barrier of nucleation using classical nucleation theory (Kashchiev, 2000 ▸; Mullin, 2001 ▸). Since the linear-chain amino acids crystallize in a layered structure, we cannot assume the nucleus of the new phase to take a spherical form. Instead we assume a cylindrical shape as schematically depicted in Fig. 7 ▸. The height of the nucleus is *r*_l_ and spans *r*_l_/*h*_l_ layers where *h*_l_ is the height of an individual layer. Within the layer we assume an isotropic circular shape of the nucleus with radius *r*, where an individual molecule covers an area *A*. The free-energy difference of such nucleus, made up of *n* molecules, with respect to the initial crystal form is 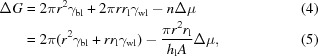
where γ_wl_ and γ_bl_ are the surface free energies within and between the layers, respectively. We define a parameter 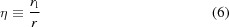
as a measure for the anisotropy in the formed nucleus. This is expected to be proportional to γ_bl_/γ_wl_ and allows us to express Δ*G* in terms of *r* and η. The critical nucleus can now be obtained by taking the derivative of Δ*G* with respect to *r* and equating this to 0, which results in 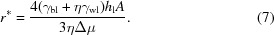
The barrier for nucleation is 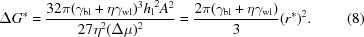
In the following, the consequences of these two quantities for the phase transitions will be discussed for two limiting cases: η ≪ 1 and η ≃ 1.

### 5.1. Transitions without torsional changes   

Even though the phase transitions described here are first order and proceed through nucleation-and-growth, they probably do not occur in a molecule-by-molecule fashion [as was proposed by Mnyukh (2010 ▸)], but in a cooperative fashion. Cooperative motion on a small length scale in these systems must not be confused with cooperative motion on an infinite length scale in higher order phase transitions, as discussed earlier.

**Figure 8:**
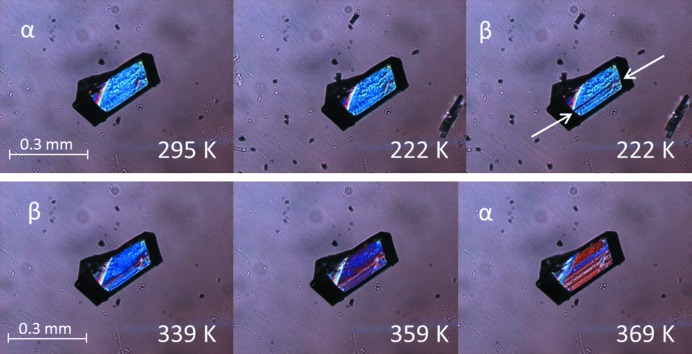
Thermal stage polarisation microscopy snapshots showing an example of a fast phase transition in some of the layers of a crystal; (top) the α → β phase transition of dl-Nle during cooling and (bottom) the β → α phase transition of dl-Nle during heating. The transforming layers are indicated by the arrows. Reprinted with permission from Smets *et al.* (2015 ▸). Copyright (2015) American Chemical Society.

The nucleus for transitions without torsional changes is typically very anisotropic as can be seen in Fig. 8 ▸, which shows thermal stage polarisation microscopy snapshots for the α ↔ β phase transition of dl-Nle. The transition occurs very rapidly within the layer, extending over the full length of the crystal. A new nucleation event needs to then occur for further transition of the crystal. This results in a very anisotropic transformed region, and we assume that the critical nucleus, which initiated this transformation, had a similar anisotropy. This shape is logical considering the anisotropy of the interactions in the crystal structure and the structural changes involved. Layers move with respect to each other without changing the layer structure itself. This is enabled by the strong hydrogen bonding within the layers and the weak van der Waals interactions between the layers. This behavior has been observed for other compounds with transitions within this class (‘pure shifts’), and hence the anisotropy parameter η can be taken as η ≪ 1 for this class. As a consequence, the critical nucleus 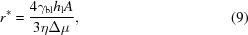
and the barrier for nucleation 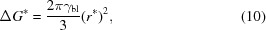
are now largely determined by the surface free energy between the layers. Empirically, we have seen that Δμ is very small for this class [see Fig. 6 ▸(*c*)]. The critical nucleus is hence rather large (both η and Δμ are small), but γ_bl_ should be small for the transition to still be possible (ΔG* not too large) as well as decreasing with chain length to explain the observed trend of decreasing transition temperature with chain length [see Fig. 5 ▸(*a*)]. It is not too surprising that γ_bl_ decreases with chain length, since longer chains are more flexible and can hence accommodate differences in relative stacking between the layers more easily. Moreover, since γ_bl_ is mostly determined by the weak interactions between the aliphatic groups at the end of the amino acid molecules, it will be relatively small. Indeed, modeling studies confirm this picture (van den Ende *et al.*, 2015 ▸, 2016 ▸). Molecular dynamics simulations of a phase transition of dl-Nle and l-Phe indicated the possibility of cooperative motion in strongly anisotropic layered structures (van den Ende *et al.*, 2015 ▸, 2016 ▸; Cuppen *et al.*, 2019 ▸). In that case, the nucleus of the new phase had a relatively small size of tens to hundreds of molecules due to the energy barriers involved. Moreover, the simulations showed that layers transform almost independently, which indicates that the 3D nucleus is very anisotropic. Nevertheless, collective displacement of molecules is possible on this length scale without breaking the hydrogen bonds between the molecules. To initiate the phase transition, 3D nucleation of the new phase is necessary, which probably occurs at defects – microcavities according to Mnyukh (2010 ▸). However, in the case of cooperative motion on a limited length scale, we expect that after 3D nucleation the subsequent propagation of the new phase within a layer of the crystal can still be hindered by defects. Naumov *et al.* also discussed that the thermosalient effect is often less pronounced in ground powders compared with single crystals, since the strain can be dissipated at defect sites and therefore does not sufficiently accumulate for collective motion (Naumov *et al.*, 2015 ▸). Defects thereby interrupt the collective displacement and slow it down dramatically, as was also shown experimentally for dl-Nle (Smets *et al.*, 2015 ▸). Nevertheless, cooperative motion can occur locally in between defects. In high-quality single crystals, fast energy transfer can take place with little dissipation, resulting in evident cooperative motion. However, the strain build-up a single crystal or crystallite can withstand before dissipating is limited. Therefore, the amino acid phase transitions involving a relatively large change in crystal structure, which generally have a large transition enthalpy, often delaminate or show cracks and the transition front propagates relatively slowly. Although the phase transitions without a torsional change generally have a large hysteresis, once the phase transition starts it propagates very fast through a layer.

### 5.2. Transitions with changes in inner torsion   

**Figure 9:**
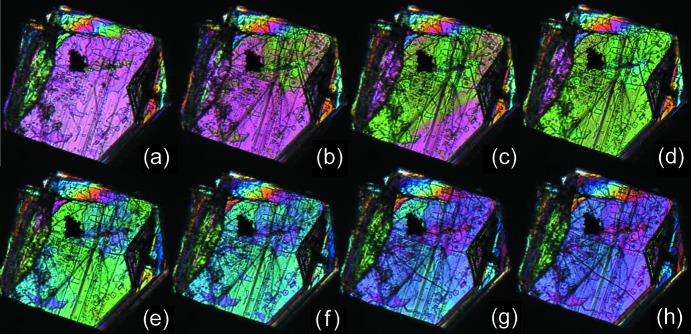
Polarisation images of a single crystal of dl-Nle (∼1 mm along the longest axis) at different temperatures during the α → γ transition. (*a*) 394.5 K (before the transition), (*b*) 394.6 K, (*c*) 394.6 K, (*d*) 394.7 K, (*e*) 394.7 K, (*f*) 394.7 K, (*g*) 394.9 K, (*h*) 394.9 K (the transition is complete).

**Figure 10:**
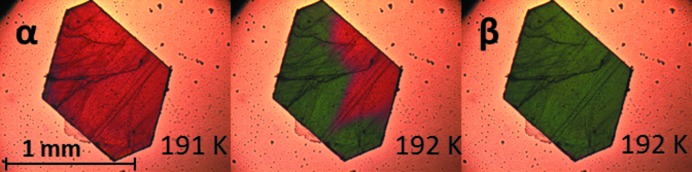
Snapshots of the dl-Nva α → β phase transition during heating, showing that the propagation fronts are parallel to the crystal facets.

For transitions with torsional changes, particularly the ‘inner torsion’ class, the picture is completely different. The layer is now distorted during the transition, although the hydrogen bonding pattern remains intact. The impact of this distortion is expected to be larger for torsion changes close to the hydrogen bonding layer. Torsional changes in one molecule can induce changes in neighboring molecules within the layer, but also in opposite layers and the critical nucleus is hence expected to be much more isotropic for this class of transitions (η ≃ 1). Thermal microscope images confirm this picture. Fig. 9 ▸ shows a heating series of a dl-Nle crystal around the α → γ transition temperature, which is a transition with an ‘inner torsion’ change. The images are taken perpendicular to the layers, but unlike transitions in the ‘pure shifts’ class, a propagation front can be observed in this direction, making the transition much more isotropic. Similar behavior is observed for the α → γ transition of dl-Nva (Fig. 10 ▸). Here the propagating front can be observed to follow the facets that are also the slowest growing planes in crystal growth. Similar propagation behavior is observed for a ferrocenyl–acetylide–gold(I) complex with triethylphosphine, where the phase-transition mechanism involves a structural change starting locally and propagating within well defined regions in the crystal lattice (Makal, 2018 ▸). Molecular dynamics simulations of structural changes that involve small changes in dihedral angles of phenyl groups also show that local fluctuations in the dihedral angle of individual molecules initiate the transformation (Duan *et al.*, 2019 ▸).

Taking η = 1, the critical nucleus size and barrier for nucleus become 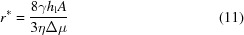
and
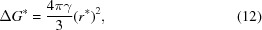
where γ is the effective isotropic surface free energy, γ = γ_wl_ = γ_bl_. Empirically, we observe a decrease in the driving force for the phase transition with chain length, this means that r* increases with chain length. Judging by the increasing transition temperature, γ increases as well. Indeed, both γ_wl_ and γ_bl_ are likely to increase with chain length. A change in one of the inner torsions will lead to large distortion, both within the layer and between the layers at the end of the chains. For longer chain lengths, the distortion is larger.

**Figure 11:**
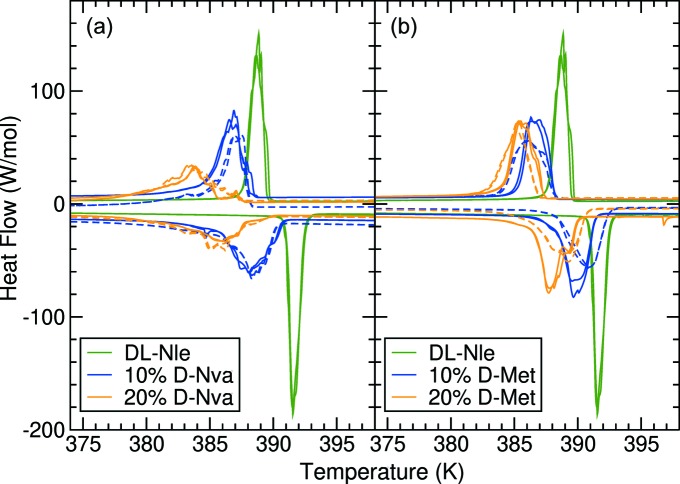
DSC diagrams of the α → γ transition in pure dl-Nle crystals (green) and crystals with 10 or 20% impurities of (*a*) d-Nva or (*b*) d-Met. Dashed and solid curves represent crystals grown from different batches and illustrate the reproducibility of the results.

A way to change the free energy within the layer is by adding impurities. Yang *et al.* (2018 ▸) showed that l-valine and l-Nva are completely miscible in the solid state. We observed that d-Nva and d-Met are miscible in dl-Nle to at least some extent and we used this to create impurities of different chain lengths in dl-Nle crystals. Fig. 11 ▸(*a*) shows the DSC of dl-Nle crystals grown from a solution where 10 or 20% of d-Nle is replaced by d-Nva. Because of the shorter chain length of d-Nva, we expect γ_wl_·*h*_l_ to be lower and to affect the transition temperature. Indeed Fig. 11 ▸(*a*) shows that the α → γ transition occurs at lower temperature, both on heating and on cooling. It also shows that the transition is more spread, with a larger temperature range. This is probably caused by the inhomogeneity in the crystal due to impurities. However, the results are in fact reproducible between crystals as can be seen by comparison of the dashed and the solid curves, which distinguish crystals grown from different batches. Impurities present in d-Met show a similar effect to that observed in Fig. 11 ▸(*b*), which shows that these types of transitions can be tailored by impurities. Impurities are typically used to bind to surfaces and block certain processes (Weissbuch *et al.*, 1991 ▸; Smets *et al.*, 2018 ▸); here, they impact on the bulk properties and hence a much larger concentration is required. For the low-temperature α ↔ β transition, which is a ‘pure shift’ transition, no significant effect could be observed, but the variability from crystal to crystal is much larger for this transition and a 2–3 K change would not be observable.

## 6. Thermosalient behavior   

**Figure 12:**
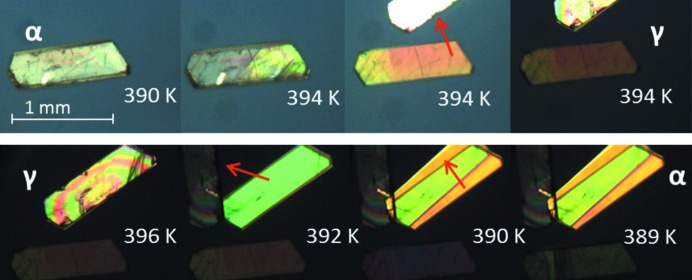
Thermal stage polarisation microscopy snapshots showing an example of jumping crystals; (top) α → γ phase transition of dl-Nle during heating and (bottom) γ → α phase transition of dl-Nle during cooling. The jumping crystal parts are indicated by the arrows.

**Figure 13:**
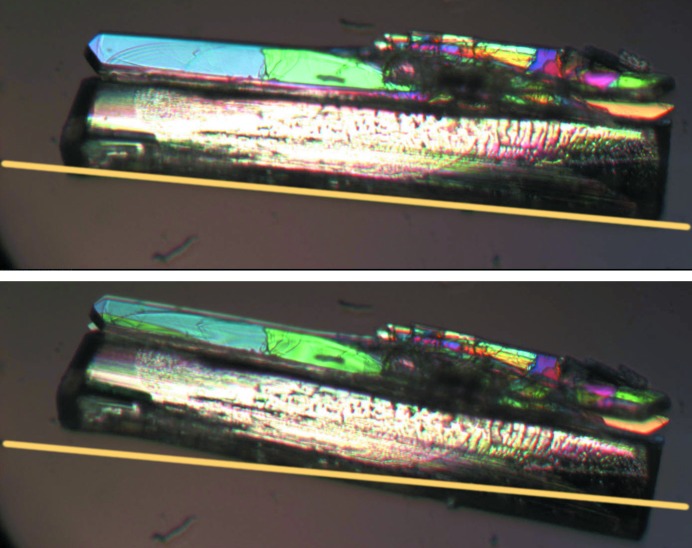
Thermal stage measurements of the α → β transition of dl-Nle. The crystal shows some rapid angular displacement (a line has been added as a reference).

Thermal stage microscopy revealed thermosalient behavior for some systems. Movements of whole crystals have been observed, but also more destructive thermosalient behavior where parts of a crystal ‘jump’ off by delamination. For instance, dl-Nle shows thermosalient behavior for both the α → β and the α → γ transition. Snapshots of the α → γ transition during heating are shown in Fig. 12 ▸, whereby clear delamination can be observed. This is known to occur irrespective of the plane on which the crystal is standing. Thermosalient behavior during the α → β phase transition is much less violent and only small displacements of the crystal can be observed (see Fig. 13 ▸). This is quite surprising since the α and β form have identical lattice parameters and hence the jump is not facilitated by an anisotropic lattice expansion between the before and after structure, but by movement and/or shape change during the transition. Moreover, the enthalpy change is very small and it is not clear that this is large enough to facilitate the mechanical response. This is therefore most likely to be associated with an entropic effect owing to the vibrational structure of the material. Similar observations have been made for tetrabromobenzene (Zakharov *et al.*, 2018 ▸).

Another surprising aspect is that the α → γ and α → β transitions belong to different classes: α → β is a ‘pure shift’ transition and α → γ an ‘inner torsion’ transition and their transition mechanisms are quite different. As mentioned previously, thermosalient behavior is typically associated with cooperative motion, but the most spectacular effects were observed for systems belonging to the ‘inner torsion’ class.

Based on the fragmented information obtained in this study we cannot draw strong conclusions on thermosalient behavior, but it does not appear to be directly linked to cooperative motion.

## 7. Conclusions   

In this work, we show that we can distinguish several types of phase transition with different characteristics in racemates and quasi-racemates of linear-chain amino acids. In general, transitions involving torsional changes proceed through a ‘classical first-order transition’, with small hysteresis, large enthalpy differences and a clear nucleation-and-growth mechanism. The transition temperature for this class increases with increasing chain length which is typical for phase transitions in aliphatic systems. Transitions with only shifts between layers behave very differently. They typically have small entropy changes, large hysteresis and their transition temperature decreases with chain length. From the data presented here, we conclude this is due to their transition mechanism which involves cooperative motion. This collective motion also requires nucleation and growth of the new phase, and should therefore not be viewed as an alternative to Mnyukh’s theory on the mechanism of phase transitions, but rather as an extension.

## 8. Experimental   

### 8.1. Materials and crystallisation   

d-2-aminobutyric acid, d-norvaline, d-norleucine, l-2-aminoheptanoic acid, d-2-amino-octanoic acid, l-2-aminooctanoic acid, d-methionine, dl-2-aminobutyric acid, dl-norvaline, dl-norleucine, dl-2-aminoheptanoic acid, dl-2-amino-octanoic acid and dl-methionine were purchased from Sigma–Aldrich and used without further purification. The racemic amino acids mentioned here were recrystallized by solvent evaporation of an aqueous solution or hanging drop crystallisation as described by Smets *et al.* (2015 ▸), and subsequently measured using DSC. Liquid-assisted grinding was performed on a Retsch Mixer Mill MM 400 at 30 Hz to form co-crystals for the following quasi-racemates in a 1:1 molar ratio (100 mg in total); d-Abu:l-Nva, d-Abu:l-Hep, d-Nva:l-Hep, d-Nle:l-Hep, d-Met:l-Hep, d-Oct:l-Hep, d-Abu:l-Oct, d-Nva:l-Oct, d-Nle:l-Oct and d-Met:l-Oct. The samples were ground in a 2.0 ml Eppendorf SafeLock microcentrifuge tube with a 0.9 mm ball for 25 min using 10 µl ethanol, and a second time for 25 min after adding another 10 µl ethanol, and subsequently measured using DSC.

### 8.2. Differential scanning calorimetry   

DSC measurements were performed using a Mettler Toledo DSC1 calorimeter with a high-sensitivity sensor (HSS8), in combination with LN2 liquid nitrogen cooling, a sample robot and *STAR^e^* software (13.00a; Mettler–Toledo, 2018 ▸). Powder samples of the racemates and the ball-milled quasi-racemates were cooled and subsequently heated in repeated cycles at a rate of 2 K min^−1^ in the temperature range 123 to 473 K. Samples of a few milligrams were sealed in an aluminium pan (40 µl) and the heat flow was measured compared with an empty reference pan as a function of temperature. The DSC was calibrated with the melting points of indium (*T*_on_ = 429.5 K and Δ_fus_ *H* = −28.13 J g^−1^) and zinc (*T*_on_ = 692.85 K and Δ_fus_ *H* = −104.77 J g^−1^), both supplied by Mettler Toledo.

### 8.3. Thermal stage polarisation microscopy   

Single crystals were studied under a nitrogen atmosphere in a Linkam LTS420 thermal stage. The thermal stage was coupled to a Zeiss Axioplan 2 Imaging polarisation microscope to observe the phase transitions *in situ* (see Figs. 8 ▸, 9 ▸, 10 ▸, 14 ▸ and 15 ▸). The microscope images were recorded with a MediaCybernetics Evolution VF digital camera. The temperature was varied between 113 and 433 K, using heating rates between 1 and 10 K min^−1^.

## Figures and Tables

**Figure 14:**
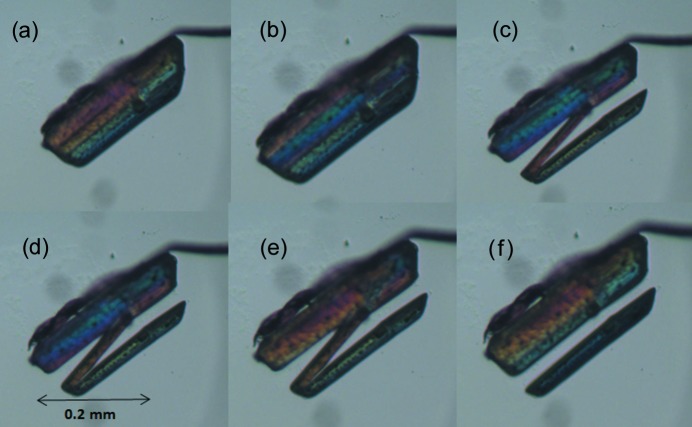
Thermal stage measurements of dl-Oct. (*a*)–(*c*) II → I transition at 442.8, 442.4 and 441.6 K, respectively. (*d*)–(*f*) I → II transition at 443.5, 444.6 and 445.6 K, respectively.

**Figure 15:**
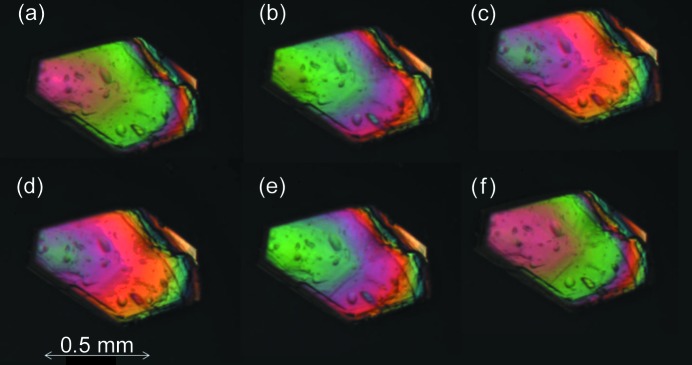
Thermal stage measurements of dl-Hep. (*a*)–(*c*) IV → V transition at 401.9, 402.4 and 403.2 K, respectively. (*d*)–(*f*) V → IV transition at 402.3, 401.1 and 399 K, respectively. Only color change can be observed.

**Table 1:** Overview of the number of enantiotropically related polymorphs known for the aliphatic linear-chain amino acid (quasi)racemates studied here

	L-Abu	L-Nva	L-Nle	L-Hep	L-Oct	L-Met
D-Abu	3	2	*1*	**2**	**3**	2
D-Nva	2	3	3	**2**	**2**	3
D-Nle	*1*	3	3	**3**	**2**	4
D-Hep	**2**	**2**	**3**	5	**2**	**3**
D-Oct	**3**	**2**	**2**	**2**	**2**	**3**
D-Met	2	3	4	3	3	2
